# Supplementary material for: Microbiome-enabled genomic selection improves prediction accuracy for nitrogen-related traits in maize
Source: G3 (Bethesda). 2023 Dec 19;14(3):jkad286. doi: 10.1093/g3journal/jkad286 (PMC11090461; doi:10.1093/g3journal/jkad286)
Supplement: jkad286_Supplementary_Data [file jkad286_supplementary_data.pdf]

Supplemental Tables

1

Table S1 Top 1% ASVs detected by the MEGS model. (https://github.com/ZhikaiYang/GP_microbiome/blob/master/data/supplementary/Supplemental_Table_S1_top_one_percent_asvs.txt)

Table S2 Mediators ASVs identified by mediation analysis. (https://github.com/ZhikaiYang/GP_microbiome/blob/master/data/supplementary/Supplemental_Table_S2_mediator_asvs.txt)

Supplemental Figures

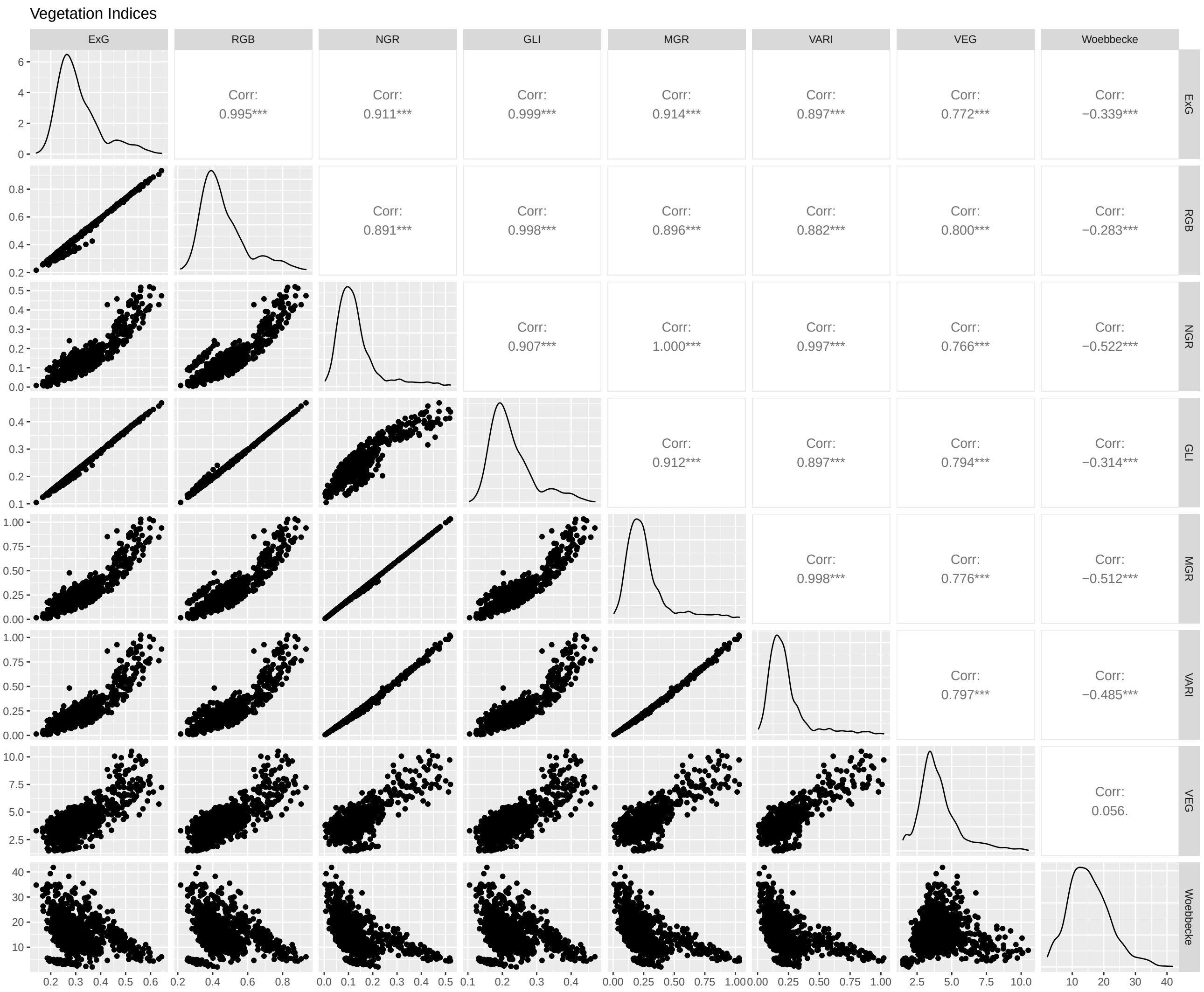

Figure S1 The distributions and correlations of the eight vegetation indices (VI). The upper right panel shows the pair-wise correlations, the lower left panel shows the corresponding scatter plots of each VI, and the diagonal shows the density plots of each VI. The statistical significance of the Pearson correlation is indicated by asterisks: * ($0.01 < \text{p-value} \leq 0.05$), ** ($0.001 < \text{p-value} \leq 0.01$), ***($0.0001 < \text{p-value} \leq 0.001$), and **** ($\text{p-value} \leq 0.0001$).

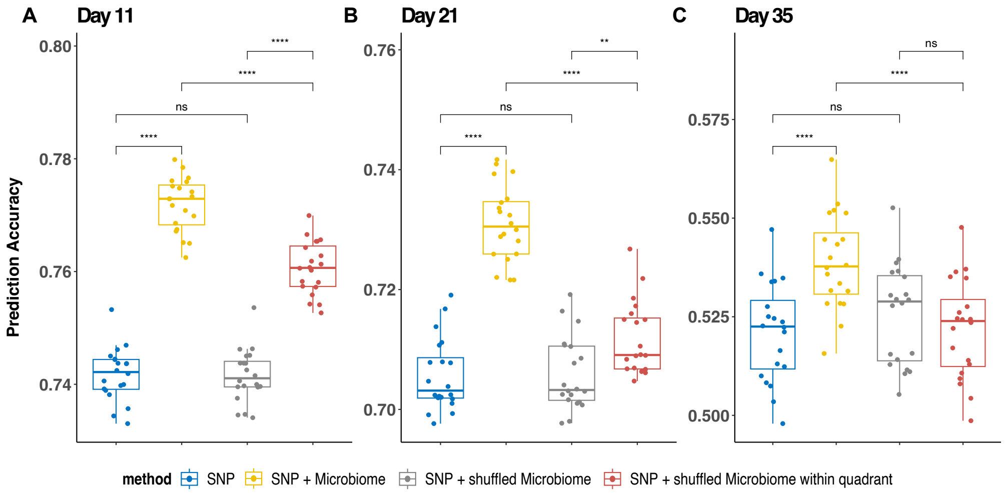

Figure S2 Comparison of prediction accuracy using different shuffling strategies. Genomic prediction results using SNPs only (blue), both SNPs and microbiome (yellow), both SNPs and shuffled microbiome (grey, shuffling within N-treatment but not within quadrant), and both SNPs and shuffled microbiome within quadrant (red). Asterisks indicate the statistical significance of the difference in accuracy between the models: ns (not significant), * (0.01 $<$ p-value $\leq$ 0.05), ** (0.001 $<$ p-value $\leq$ 0.01), ***(0.0001 $<$ p-value $\leq$ 0.001), and **** (p-value $\leq$ 0.0001).

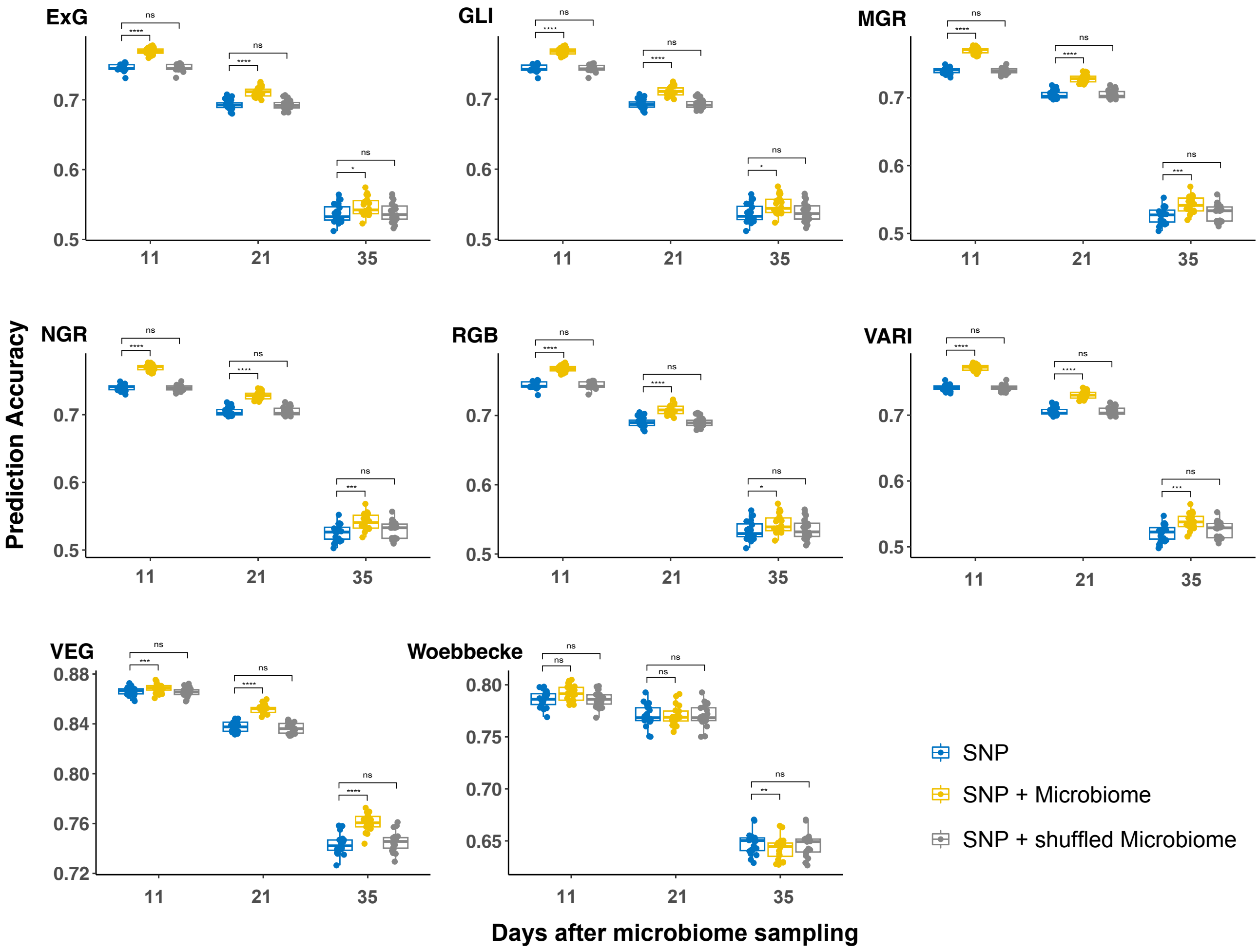

Figure S3 Prediction accuracy of VIs by incorporating microbiome into the prediction model. Genomic prediction results using SNPs only (blue), both SNPs and microbiomes (yellow), and both SNPs and shuffled microbiomes (grey). The statistical significance of the difference in accuracy between the models is indicated by asterisks: ns (not significant), * (0.01 $<$ p-value $\leq$ 0.05), ** (0.001 $<$ p-value $\leq$ 0.01), ***(0.0001 $<$ p-value $\leq$ 0.001), and **** (p-value $\leq$ 0.0001).

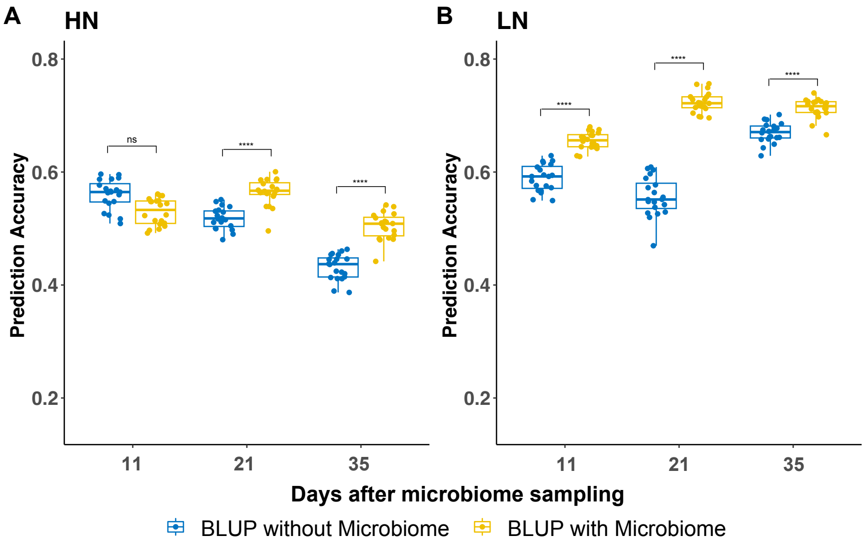

Figure S4 Prediction accuracy of incorporating microbiome under different N conditions. The prediction accuracy of model 1: getting BLUP excluding microbiome effects (blue) and model 2: getting BLUP including microbiome effects (yellow) in high N (**A**) and low N (**B**) conditions. The statistical significance of the difference in accuracy between the models using one tailed t-test is indicated by asterisks: ns (not significant), * ($0.01 < $ p-value ≤ 0.05), ** ($0.001 <$ p-value ≤ 0.01), ***($0.0001 <$ p-value ≤ 0.001), and **** (p-value ≤ 0.0001).

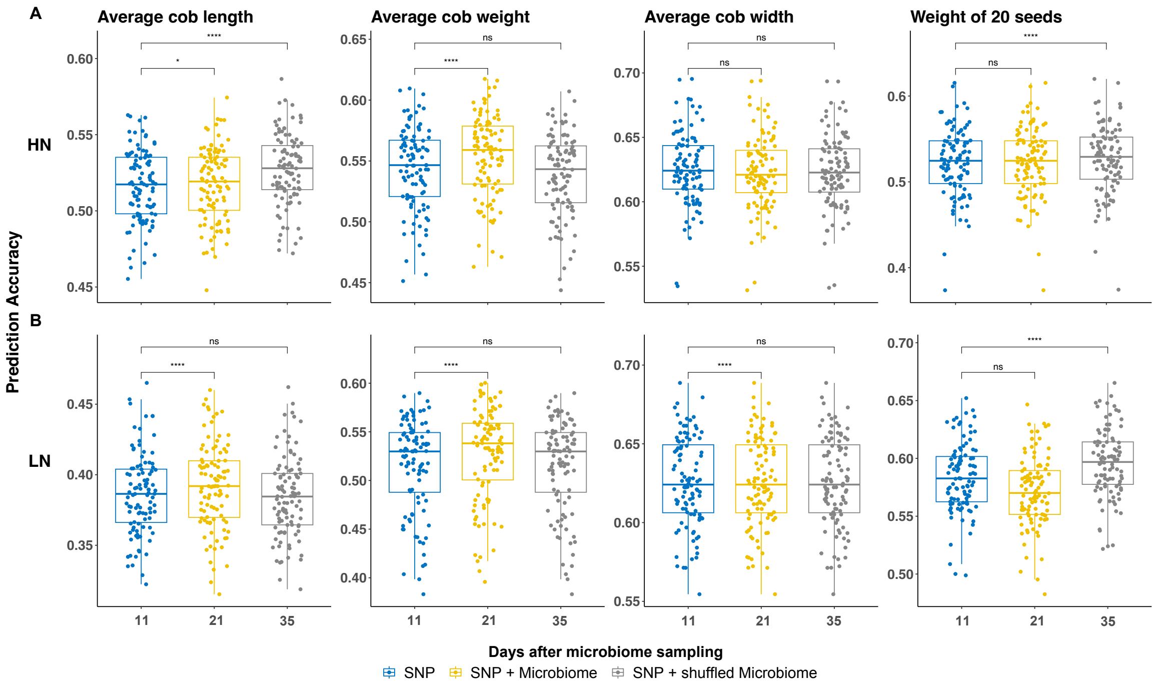

Figure S5 Prediction accuracy for yield-related traits under different N conditions. Genomic prediction results using SNPs only (blue), both SNPs and microbiomes (yellow), and both SNPs and shuffled microbiomes (grey) in the case of high N (**A**) and low N (**B**) conditions. Asterisks indicate the statistical significance of the difference in accuracy between the models: ns (not significant), * ($0.01 <$ p-value ≤ 0.05), ** ($0.001 <$ p-value ≤ 0.01), *** ($0.0001 <$ p-value ≤ 0.001), and **** (p-value ≤ 0.0001).

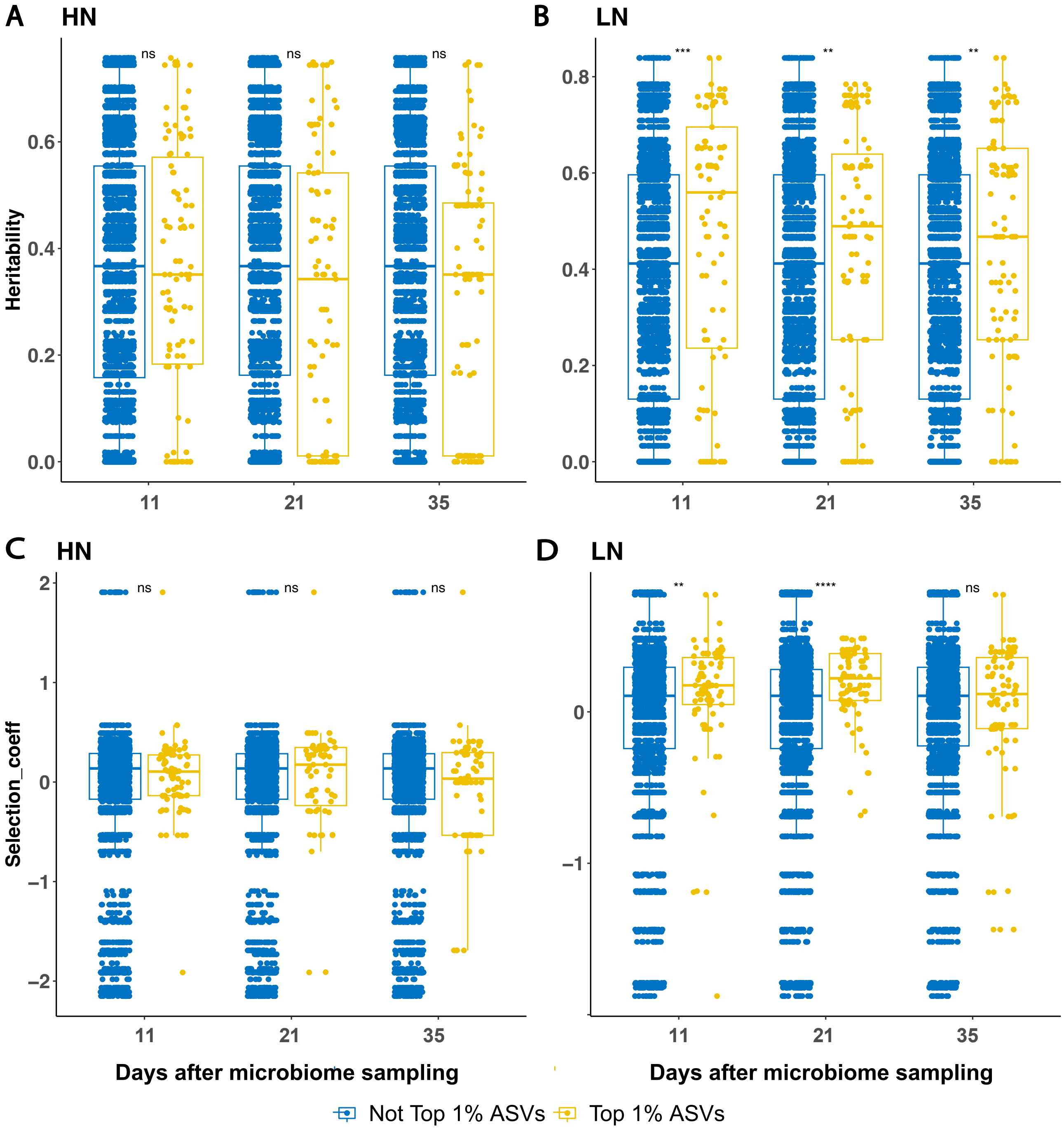

Figure S6 Comparison between top 1% ASVs and the remaining ASVs for heritability and selection coefficient. The Wilcoxon rank sum and signed rank test results between top 1% ASVs (yellow) and the remaining 99% ASVs (blue) for heritability under HN (**A**) and LN (**B**), and selection coefficient under HN (**C**) and LN (**D**), respectively. The statistical significance of the difference of the Wilcoxon rank sum and signed rank test result is indicated by asterisks: ns (not significant), * ($0.01 < \text{p-value} \leq 0.05$), ** ($0.001 < \text{p-value} \leq 0.01$), ***($0.0001 < \text{p-value} \leq 0.001$), and **** ($\text{p-value} \leq 0.0001$).

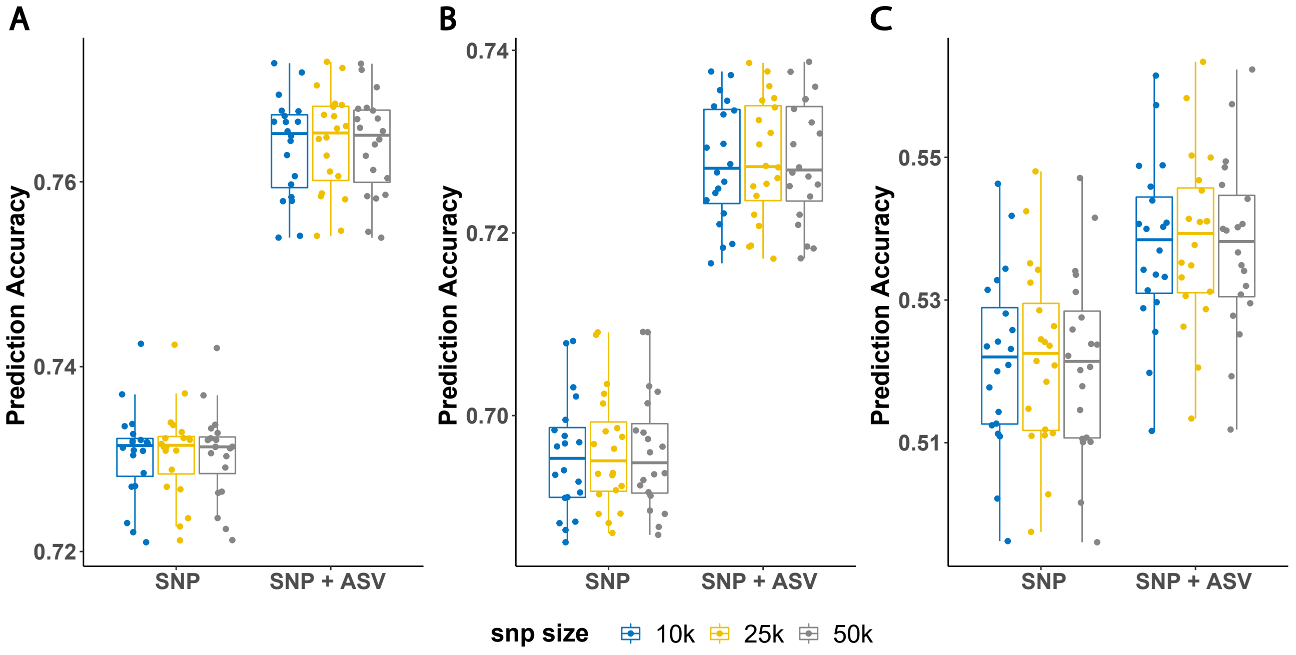

Figure S7 Sensitivity test of the SNP size on prediction accuracy for VARI trait. The Student's t-test results using randomly selected 10k SNPs (blue), 25k SNP (yellow), and 50k SNP (grey) in 11 (**A**), 21 (**B**) and 35 (**C**) days after microbiome sampling. The test results are not significant with p-values greater than 0.1.
